# High performance photocatalyst TiO_2_@UiO-66 applied to degradation of methyl orange

**DOI:** 10.1186/s11671-023-03894-6

**Published:** 2023-09-11

**Authors:** Jingyi Yang, Xue Chang, Fang Wei, Zixiao Lv, Huiling Liu, Zhan Li, Wangsuo Wu, Lijuan Qian

**Affiliations:** 1 School of Nuclear Science and Technology, Lanzhou University, Lanzhou, 73000 People’s Republic of China; 2 Frontiers Science Center for Rare Isotopes, Lanzhou University, Lanzhou, 730000 China; 3 Key Laboratory of Special Function Materials and Structure Design, Ministry of Education, Lanzhou, 730000 Gansu China

## Abstract

MOFs have considerable adsorption capacity due to their huge specific surface area. They have the characteristics of photocatalysts for their organic ligands can absorb photons and produce electrons. In this paper, the photodegradation properties of TiO_2_ composites loaded with UiO-66 were investigated for the first time for MO. A series of TiO_2_@UiO-66 composites with different contents of TiO_2_ were prepared by a solvothermal method. The photocatalytic degradation of methyl orange (MO) was performed using a high-pressure mercury lamp as the UV light source. The effects of TiO_2_ loading, catalyst dosage, pH value, and MO concentration were investigated. The results showed that the degradation of MO by TiO_2_@UiO-66 could reach 97.59% with the addition of only a small amount of TiO_2_ (5 wt%). TiO_2_@UiO-66 exhibited significantly enhanced photoelectron transfer capability and inhibited efficient electron–hole recombination compared to pure TiO_2_ in MO degradation. The composite catalyst indicated good stability and reusability when they were recycled three times, and the photocatalytic reaction efficiencies were 92.54%, 88.76%, and 86.90%. The results provide a new option to design stable, high-efficiency MOF-based photocatalysts.

**Supplementary Information:**

The online version contains supplementary material available at 10.1186/s11671-023-03894-6.

## Introduction

With the rapid development of industry, human activities have caused a lot of environmental pollution [1]. Among the environment wastewater, toxic organic pollutants have serious impacts on ecosystems and human health. Thus, many researchers have made great efforts to develop efficient treatment technologies to remove pollutants in water [2]. Conventional methods for the removal of organic pollutants include adsorption [3], precipitation [4], membrane separation, and biological treatments [5]. Among various physical and chemical pollutant removal methods, photocatalytic degradation of organic pollutants has been proved to be a simple, cost-effective, and environmentally-friendly method [6, 7]. The photocatalysis is the redox reaction of the semiconductor material with the reactant under the irradiation of light. Researchers have found that a variety of semiconductor materials such as TiO_2_, Fe_2_O_3_, WO_3_, CdS, Bi_2_WO_6_, BiOCl, g-C_3_N_4_, and MOFs can be used for the degradation of organic pollutants, hydrogen production from water decomposition, organic synthesis, and heavy metal ion reduction [8–13].

Metal–organic frameworks (MOFs) have attracted great interest from researchers all over the world due to their unique, outstanding properties, and potential applications [14, 15]. Among various MOFs, Zr-based MOFs (UiO-66) have gained crucial interest in photoinduced water treatment due to their construction of Zr_6_O_4_(OH)_4_ clusters and organic linkers [14]. UiO-66 has high thermal stability, superior chemical resistance to a variety of solvents, excellent chemical stability under a variety of conditions, and excellent corrosion resistance to high external pressure. Theoretical studies show that when UiO-66 is exposed to light, the organic ligands are excited to generate electrons, and then the electrons are transferred to the metal centers through valence bonds, that is, the ligand–metal charge transfer occurs, which effectively increases the lifetime of photogenerated carriers [16–18]. UiO-66 can be directly used as a photocatalyst or subjected to various modifications. The common photocatalytic modifications of UiO-66 include the preparation of UiO-66 with defects, the preparation of functionalized UiO-66-NH_2_, the incorporation of other metal ions other than Zr, the modification of materials with specific properties after synthesis, and the formation of composite materials after mixing with other substances. Among them, the last modification is often used because of its simply preparation method. The hybrid nanoparticles, such as magnetic nanoparticles, alumina, silica, graphene oxide (GO), carbon nanotubes (CNTs), polymers and amorphous carbon and other suitable materials has received much attention [14]. Combining UiO-66 nanoparticles with these nanoparticles can improve the physicochemical properties, external morphology, adsorption kinetics, and stability of MOF. This approach has two advantages applied in photocatalysis: (i) the combined coverage of the light absorption edge and visible region is expanded compared to UiO-66 or its amino-functionalized derivative (UiO-66-NH_2_), resulting in a narrower band gap of the photocatalyst; (ii) improved separation efficiency of photogenerated electron–hole pairs due to electron transfer between UiO-66 and semiconductor.

TiO_2_ photocatalysts have been widely concerned by researchers due to their low price, good chemical stability, and high photocatalytic efficiency [19–21]. However, their performance is hindered by poor adsorption properties and rapid recombination of photogenerated carriers. To alleviate these problems, many efforts have been made, such as morphological design [22], elemental doping [23], and heterojunction engineering [24]. It is recognized that an effective combination of multiple strategies will maximize the efficiency of photocatalysts.

The coupling of TiO_2_ and UiO-66 (or UiO-66-NH_2_) has gained attention in photocatalysis. TiO_2_@UiO-66 was used for the degradation of rhodamine B (RhB) and methylene blue (MB) [25], dimethyl sulphide [26], catalytic reduction of CO_2_ to CH_4_ [27], and degradation of volatile organic compounds [21]. TiO_2_@UiO-66-NH_2_ was used in the degradation of toluene, ether [28], bisphenol A and the reduction of Cr(VI) [29]. Ternary catalyst containing TiO_2_ and UiO-66 (or UiO-66-NH_2_), such as UiO-66-FP/HPW/TiO_2_ used in catalytic reduction of CO_2_ to CH_4_ [30], degradation of RhB studied by TiO_2_/UiO-66/GO [31], and ketoprofen by C-dots/TiO_2_ NS/UiO-66-NH_2_ [32].

The degradation of anionic organic dyes MO by pure UiO-66 has been studied [33–36], while the degradation of cationic dyes, such as RhB, by UiO-66-NH_2_, has also been studied [31, 37]. Also there have been studies on the degradation of MO by α-Fe_2_O_3_, CdS, In_2_S_3_ mixed with UiO-66 or UiO-66-NH_2_. In contrast, the degradation of MO by TiO_2_ compound UiO-66 has not been studied, it is necessary to study TiO_2_@UiO-66 as a catalyst to determine the effect of TiO_2_ addition on the degradation of MO by UiO-66 for anionic organic dyes.

**Fig. 1:**
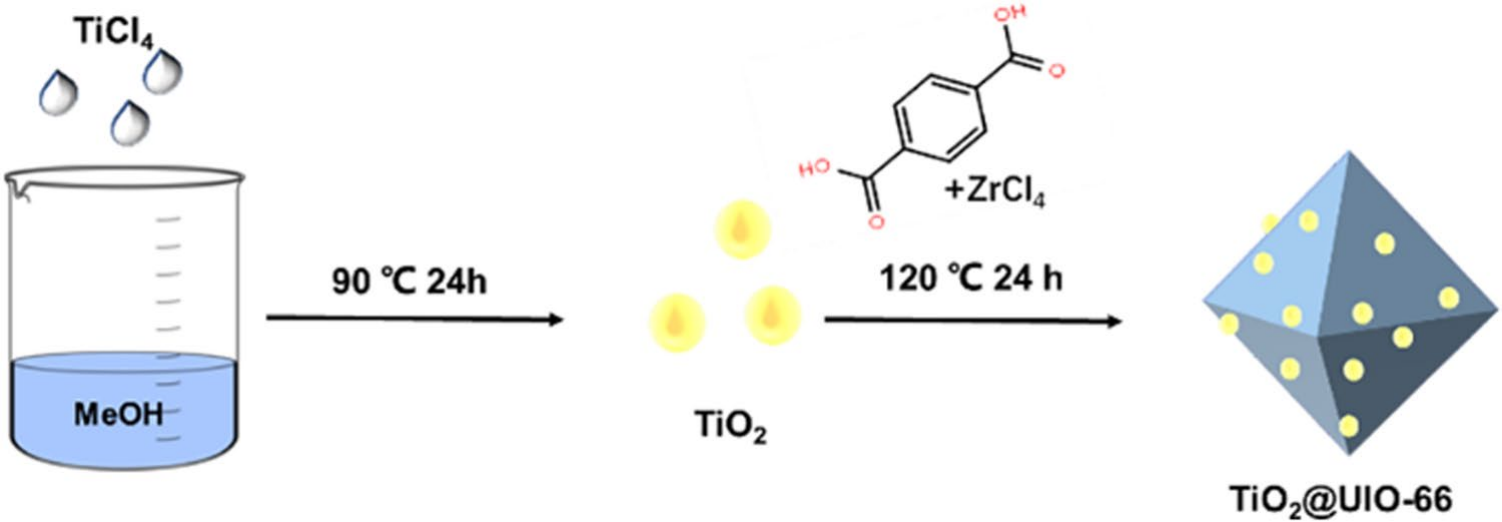
Synthesis process of TiO_2_@UiO-66

Here, TiO_2_@UiO-66 was successfully prepared by a simple solvothermal method (Fig. 1). The photocatalytic activity of MO was evaluated by UV-light degradation. TiO_2_@UiO-66 exhibits stronger photocatalytic activity than TiO_2_ and UiO-66 under UV light. In addition, the composite catalyst also showed good stability and reusability.

## Materials and methods

### Chemicals

1,4-Benzenedicarboxylic acid (BDA) (C_8_H_6_O_4_, 98%) was purchased from Macklin. Spin trap 5,5-dimethyl-1-pyroline N-oxide (DMPO, 99%) was obtained from Sigma. Zirconium chloride (IV) (ZrCl_4_, 98%), acetic acid (HAc, 99%), titanium tetrachloride (TiCl_4_, 99%), ethanol (C_2_H_5_OH, 99.7%) and dimethylformamide (DMF, 99%) were obtained from Xilong Scientific Co., Ltd. Moreover, the chemicals used in the present study were analytical grade and utilized as received.

### Synthesis of photocatalysts

#### Synthesis of TiO_2_

TiO_2_ was synthesized according to the reported method with little modification [38]. Typically, 10 mL TiCl_4_ was added to 25 mL of methanol slowly at 0 °C in an ice-water bath. Then the yellow solution was transferred to a 50 mL Teflon-lined autoclave at 90 °C for 24 h. After cooling down to room temperature, the white powder was washed with hexane/methanol (1:3, v/v) three times. The resulting power was re-suspended in hexanes and centrifuged twice. The obtained TiO_2_ was dried at room temperature.

#### Synthesis of TiO_2_@UiO-66

Dissolving ZrCl_4_ (160 mg) and BDA (114 mg) was in 26 mL DMF, then a certain quality of TiO_2_ was added to the mixture. The obtained mixture was sealed and placed in a preheated oven at 120 °C for 24 h. The product was isolated by centrifugation and rinsed with DMF and MeOH. Finally, TiO_2_@UiO-66 was dried at 60 °C overnight. TiO_2_@UiO-66(1), TiO_2_@UiO-66(3), TiO_2_@UiO-66(5), and TiO_2_@UiO-66(7) mean that the load of TiO_2_ is 1%, 3%, 5%, and 7%.

### Materials characterization

Powder X-ray diffraction (XRD) patterns were determined on a SmartLab (Ultima IV, Rigaku Corporation, Japan) with Cu-Ka radiation and a scan rate of 10°·min^−1^ between $5^\circ$ and $80^\circ$. Thermogravimetric Analysis (TGA) data was obtained from 25 to 800 °C by a Linseis STA PT 1600 ((Linseis Messgeräte GmbH, Germany). The morphology of the photocatalysts was measured by transmission electron microscope (TEM, Tecnai F30) and scanning electron microscope (SEM, Apreo S). The Fourier-transform infrared (FT-IR) spectra of the photocatalysts was recorded using a NEXUS 670 in the wavelength range of 400–4000 cm^−1^. Specific surface area, pore size distribution and pore volume of all samples tested were determined using a Micromeritics ASAP2020 apparatus. And the surface area was calculated using the Brunauer–Emmett–Teller technique, whilst the pore size distribution was measured from the nitrogen isotherms using Barrett-Joyner-Halenda (BJH) method and the Horvath-Kawazoe method, respectively. The UV–Vis diffuse reflectance spectra of the samples were collected with a UV–Vis spectrometer (Lambda 950 + Refle, spectral range 250–800 nm). The photoluminescence spectra (PL) were surveyed on an FLS920 spectrophotometer. Electrochemical impedance testing using an electrochemical workstation (CHI 660E made by Chenhua, Shanghai, with Pt as counter electrode and Ag/AgCl electrode as reference electrode). The generated radicals under 250 W high-pressure mercury lamp illumination were measured with the electron spin resonance (ESR) spectrometer (Bruker A300-10/12).

### Measurement of photocatalytic activities

100 mL MO solution (15 mg·L^−1^) was mixed with a certain amount of catalyst before UV irradiation, the suspension was stirred for 60 min in the dark to reach adsorption–desorption equilibrium. After that, the suspensions were irradiated by UV light from a 250 W high-pressure mercury lamp. 2 mL of the suspension was sampled every 30 min. The suspension was centrifuged to remove the solid catalyst, then define the degradation rate: X% = (1− C_t_/C_0_) × 100%, where C_0_ was the initial concentration of solution and Ct was the solution concentration for each period.

## Results and discussion

### Characterization

**Fig. 2:**
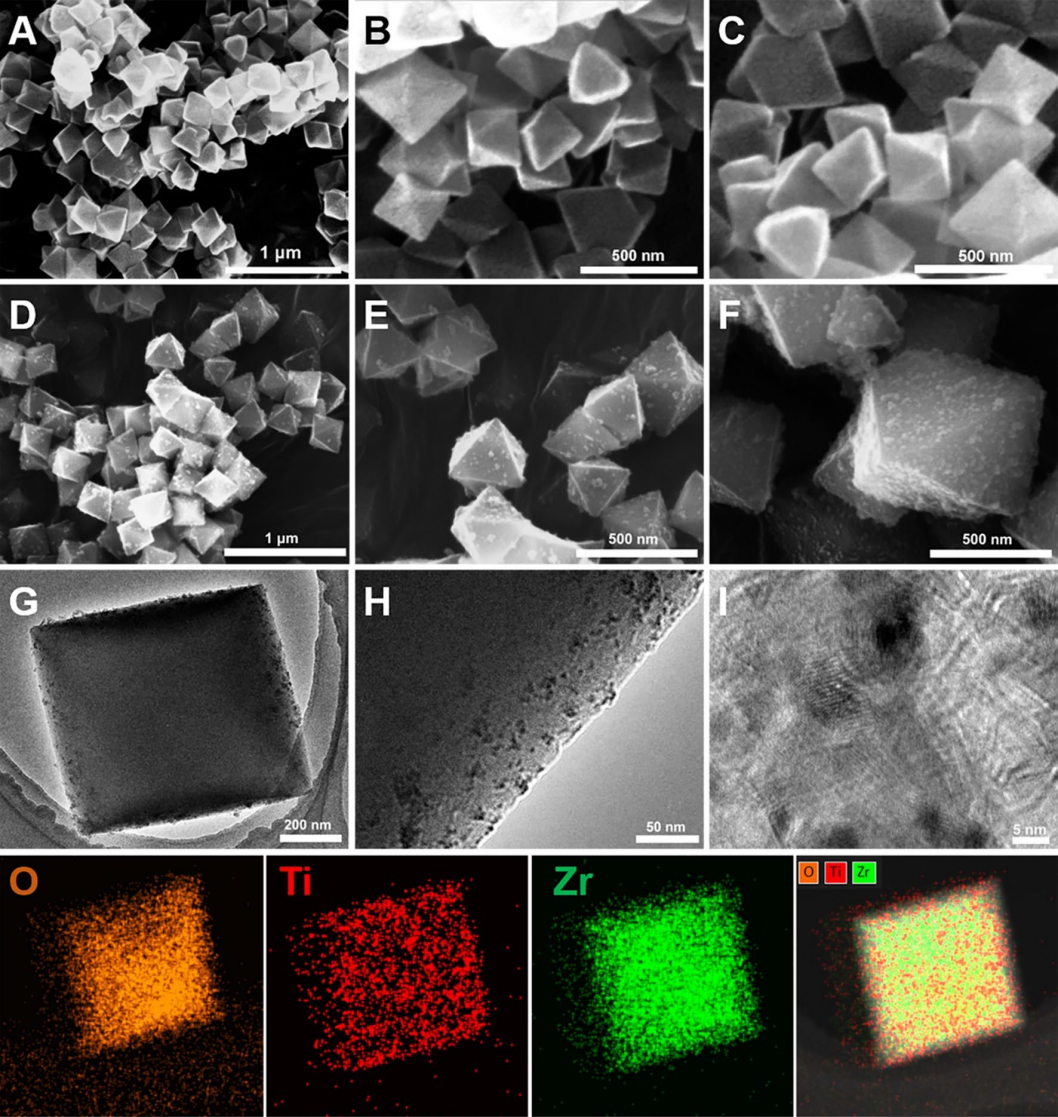
The morphological characteristics of TiO_2_@UiO-66. **A**–**C** SEM images of UiO-66; **B**–**F** SEM images of TiO_2_@UiO-66(5); (G-I) TEM images of TiO_2_@UiO-66(5) and Elemental mapping images of O, Ti, Zr elements in TiO_2_@UiO-66(5) in last line

SEM of the surface morphology of UiO-66 and TiO_2_@UiO-66 is presented in Fig. 2A-F. It can be seen that the as-synthesized pristine UiO-66 exhibits a smooth, regular, and uniform octahedral appearance with a size of about 300 nm, the same as the shapes obtained by solvothermal reactions in other literature [39, 40]. Compared with UiO-66, the size and morphology of TiO_2_@UiO-66 composite did not change significantly. The difference is that uniformly distributed TiO_2_ nanoparticles are found on the surface of TiO_2_@UiO-66. From the above observations, it can be inferred that adding TiO_2_ to the precursor solution of UiO-66 does not affect the growth of UiO-66. TEM images of TiO_2_@UiO-66 are shown in Fig. 2G–I. It can be observed that TiO_2_@UiO-66 composite has a regular octahedral shape, and its surface is evenly distributed with TiO_2_ nanoparticles, which can effectively alleviate the agglomeration of TiO_2_ during the reaction process and increase the point of the active site, and can promote the separation of carriers. According to the results of the Mapping maps, we can see that Ti, O, and Zr elements are uniformly distributed in TiO_2_@UiO-66.

**Fig. 3:**
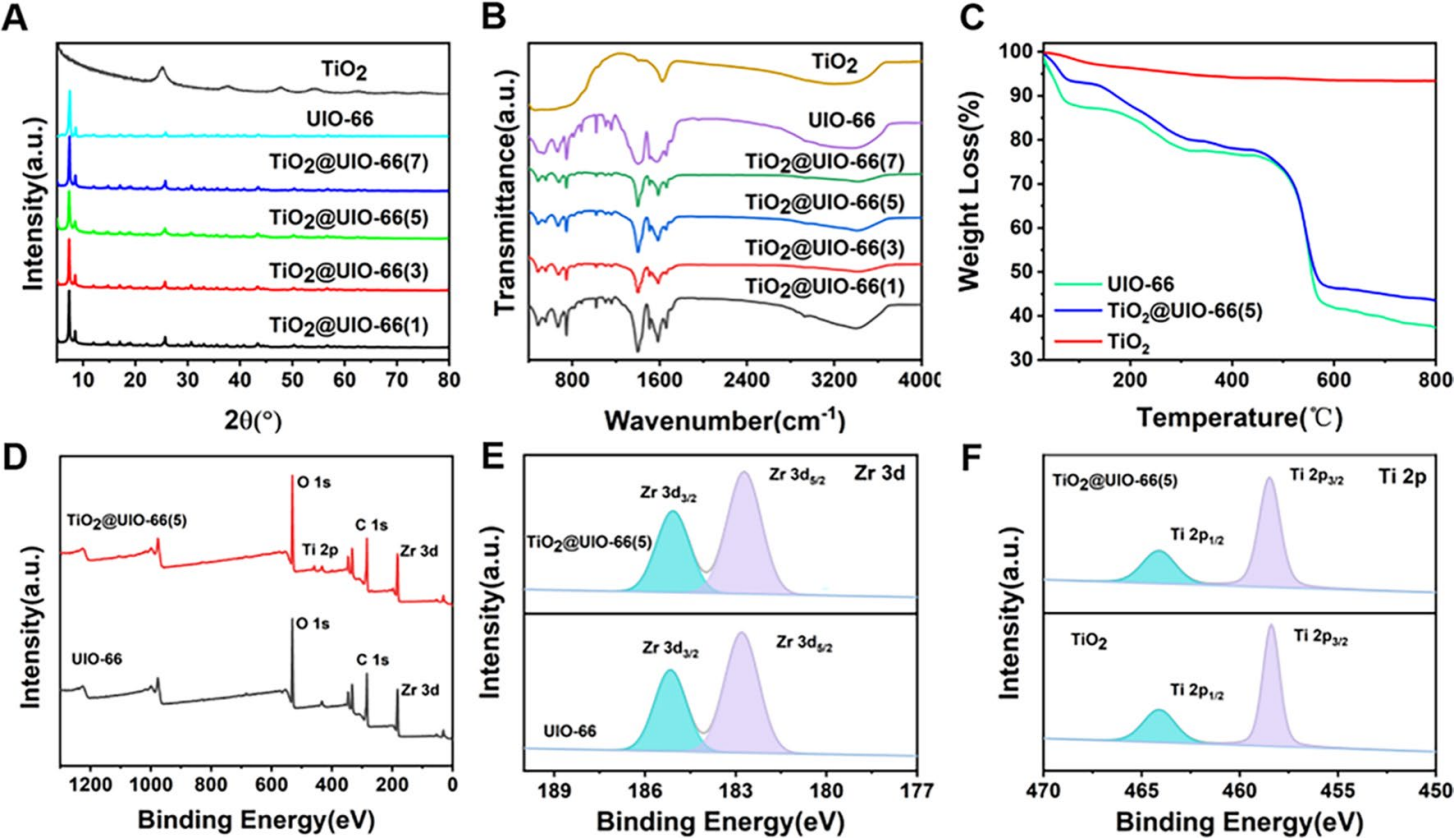
The characterizations of TiO_2_@UiO-66. **A** X-ray diffraction patterns of UiO-66, TiO_2_ and TiO_2_@UiO-66 composite materials; **B** FT-IR spectra of UiO-66, TiO_2_ and TiO_2_@UiO-66 composite materials; **C** TG spectra of UiO-66, TiO_2_ and TiO_2_@UiO-66(5) composite materials; (D-F) The XPS spectra for TiO_2_ and TiO_2_@UiO-66(5) composite materials

The XRD pattern of UiO-66 (Fig. 3A) is matched with that reported in the literature [41], demonstrating UiO-66 has been successfully synthesized. In the case of TiO_2_ (Fig. 3A), the typical diffraction peak of anatase TiO_2_ (JCPDS 21–1272) suggests that anatase TiO_2_ was prepared. As for TiO_2_@UiO-66(1), TiO_2_@UiO-66(3), TiO_2_@UiO-66(5), and TiO_2_@UiO-66(7) composites, no obvious TiO_2_ diffraction peaks were found in the XRD patterns, which should be attributed to the low amount of TiO_2_ in TiO_2_@UiO-66. Figure 3B are FT-IR spectra of TiO_2_, UiO-66, and TiO_2_@UiO-66 composites. The FT-IR spectrum of TiO_2_ indicated that the broad peak located at 400–900 cm^−1^ corresponds to the Ti–O-Ti stretching vibration peak [42]. In the spectrum of UiO-66, the peaks at 1403 and 1564 cm^−1^ are assigned to the asymmetric and symmetric vibrations of C = O on BDA, respectively [43, 44]; the peak at 537 cm^−1^ is related to the Zr-(OC) asymmetric stretching vibration [45].

The asymmetric stretching vibrations at 663 and 507 cm^−1^ are attributed to the µ_3_-O and µ_3_-OH stretching vibrations. Compared with pristine UiO-66, TiO_2_@UiO-66 show a significant decrease in the relative intensity of the characteristic peaks of UiO-66 could be observed, which could be attributed to the strong interaction between TiO_2_ and UiO-66 [21].

Figure 3C shows the TG analysis of UiO-66, TiO_2_@UiO-66(5), and TiO_2_. UiO-66 and TiO_2_@UiO-66(5) show similar weight loss processes. The weight loss at 50–150 °C in the first stage is mainly due to the evaporation of water on the surface of the material. The weight loss in the second stage in the range of 200–400 °C is due to the evaporation of DMF in the pores of UiO-66. The weight loss in the third stage in the range of 450–600 °C is mainly due to the decomposition of BDA [28]. Therefore UiO-66 has high thermal stability, which is consistent with previous reports [40]. It should be noted that the thermal stability of the TiO_2_@UiO-66 composite is higher than that of the pristine UiO-66. It is not difficult to see from the TG results that UiO-66, TiO_2_@UiO-66, and TiO_2_ exhibit relatively high thermal stability.

The elemental composition and chemical state of composite photocatalysts were explored using XPS. Figure 3D shows the full XPS spectra of UiO-66 and TiO_2_@UiO-66, where the TiO_2_@UiO-66 composite is mainly composed of C, Zr, O, and Ti elements. In UiO-66, the peaks with binding energies at 182.9 and 185.3 eV are Zr 3d_5/2_ and Zr 3d_3/2_, respectively [46, 47], while the Zr 3d in TiO_2_@UiO-66 is shifted to 182.7 and 185.0 eV, indicating that the introduction of TiO_2_ affects the chemical environment of UiO-66 (Fig. 3E). Figure 3F shows the high-resolution spectrum of Ti 2p of TiO_2_@UiO-66. The peaks at the binding energy of 458.49 and 464.14 eV are assigned to the Ti 2p_3/2_ and Ti 2p_1/2_ [48], which are similar to those of TiO_2_. Ti 2p_3/2_ shifts to lower binding energy, which means that the successful combination of TiO_2_ and UiO-66 is favorable for electron transfer between UiO-66 and TiO_2_.

**Fig. 4:**
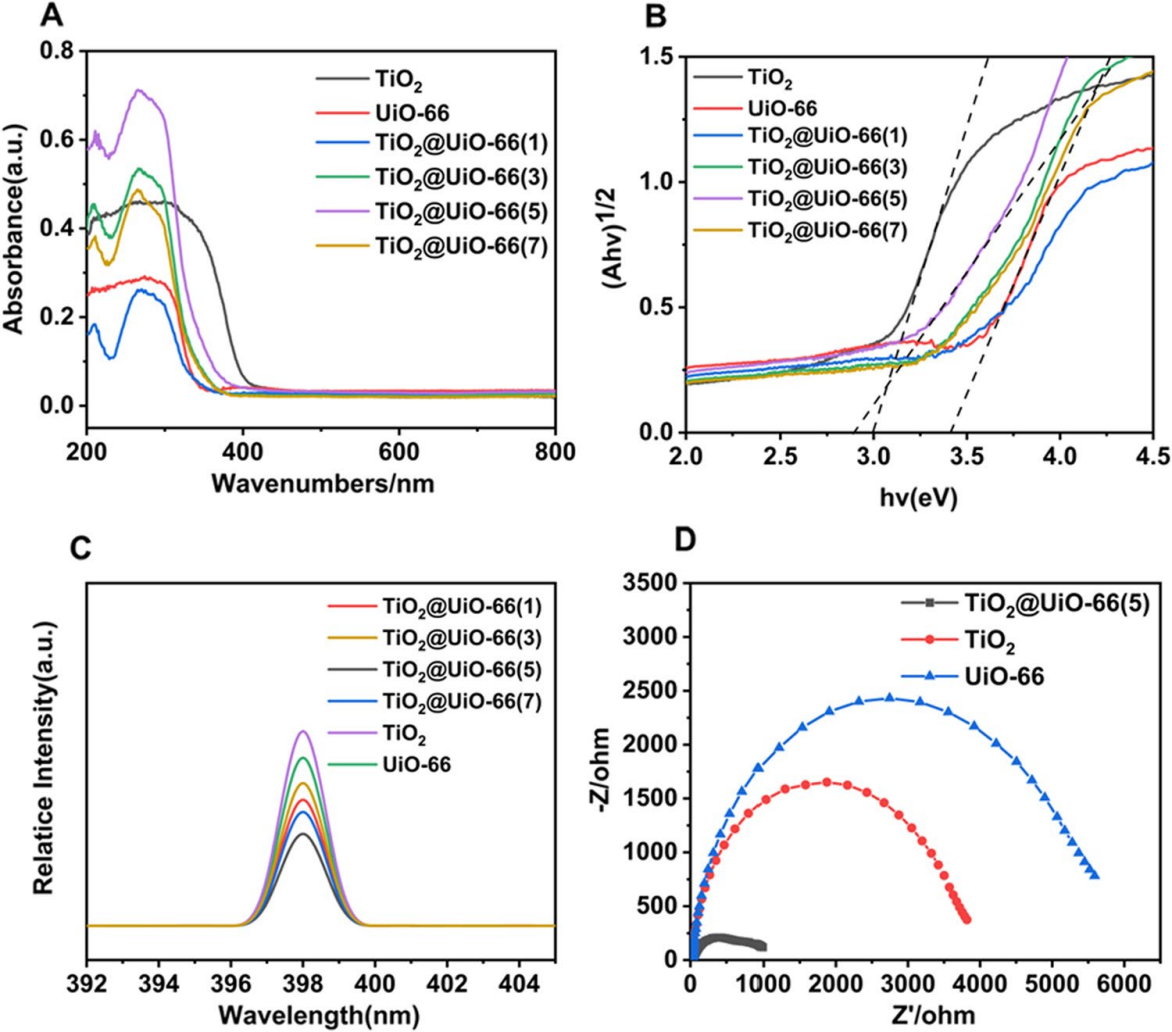
The optical characterization of TiO_2_@UiO-66. **A** UV–vis spectroscopy for the composite materials; **B** The converted Tauc plot of (αhν)^2^ versus photo energy; **C** The PL spectra of the composite materials; **D** The EIS spectra of TiO_2_, UiO-66 and TiO_2_@UiO-66(5) composite materials

The spectra of UV–visible light absorption (Fig. 4A) were obtained to be evaluated light-absorption ability. The UV light region absorption intensity of TiO_2_@UiO-66(5) is much higher than other samples. This is expected to improve the photocatalytic performance of TiO_2_@UiO-66 composite for MO degradation.

To determine the band energy diagram of the TiO_2_@UiO-66 heterojunction, the valence band (VB) maximum and band gap energy (Eg) of pure TiO_2_ and UiO-66 have been investigated. Figure 4B presents the plot between (αhν)^2^ and photon energy for band gap determination. According to the Kubelka-Munke equation, the Eg of these samples can be obtained [49]^.^ TiO_2_, UiO-66 and TiO_2_@UiO-66(5) have the Eg of 3.00, 3.41 and 2.90 eV, respectively. The lowering of the band gap is used to improve the macroscopical solar energy utilization of MOF-based photocatalysts. Thus, the decrease in Eg for the TiO_2_@UiO-66(5) heterostructure could be advantageous for the enhanced photocatalytic performance by increasing light-harvesting efficiency, theoretically.

Figure 4C reveals the photoluminescence spectrum of TiO_2_, UiO-66, and TiO_2_@UiO-66 samples under 300 nm excitation wavelength. When compared with TiO_2_, the PL intensity of TiO_2_@UiO-66 declined. The lower recombination rate of photogenerated charge careers led to the reduction of PL intensity, suggesting that the TiO_2_@UiO-66(5) exhibits a better property to the separation of electron–hole pair [50]. The separation and transport efficiency of photogenerated carriers are the decisive factors affecting the photocatalytic process. EIS patterns (Fig. 4D) show that the as-prepared materials are arranged in the following order: UiO-66 > TiO_2_ > TiO_2_@UiO-66(5), which means that the recombination of UiO-66 with TiO_2_ nanoparticles will facilitate electron transfer [51].

The specific surface area and pore size distribution of UiO-66 and TiO_2_@UiO-66 composites were determined by the N_2_ adsorption method. Figure S1 show that the adsorption–desorption isotherms of both materials are type I isotherms, and the TiO_2_@UiO-66 composite and UiO-66 are typical microporous materials. The calculated BET surface area and pore size of UiO-66 and TiO_2_@UiO-66 are shown in Table S1. The BET surface areas of UiO-66 and TiO_2_@UiO-66(5) are 775.12 m^2^·g^−1^ and 685.94 m^2^·g^−1^, respectively, and the pore sizes are 2.45 nm and 1.98 nm, respectively. According to the results in Table S1, it can be concluded that when UiO-66 is compounded with TiO_2_, the specific surface area, and pore size decrease, indicating that the introduction of TiO_2_ blocks part of the pores of UiO-66, but still has a large specific surface area.

### Photocatalytic performance

#### Effects of TiO_2_ loading

**Fig. 5:**
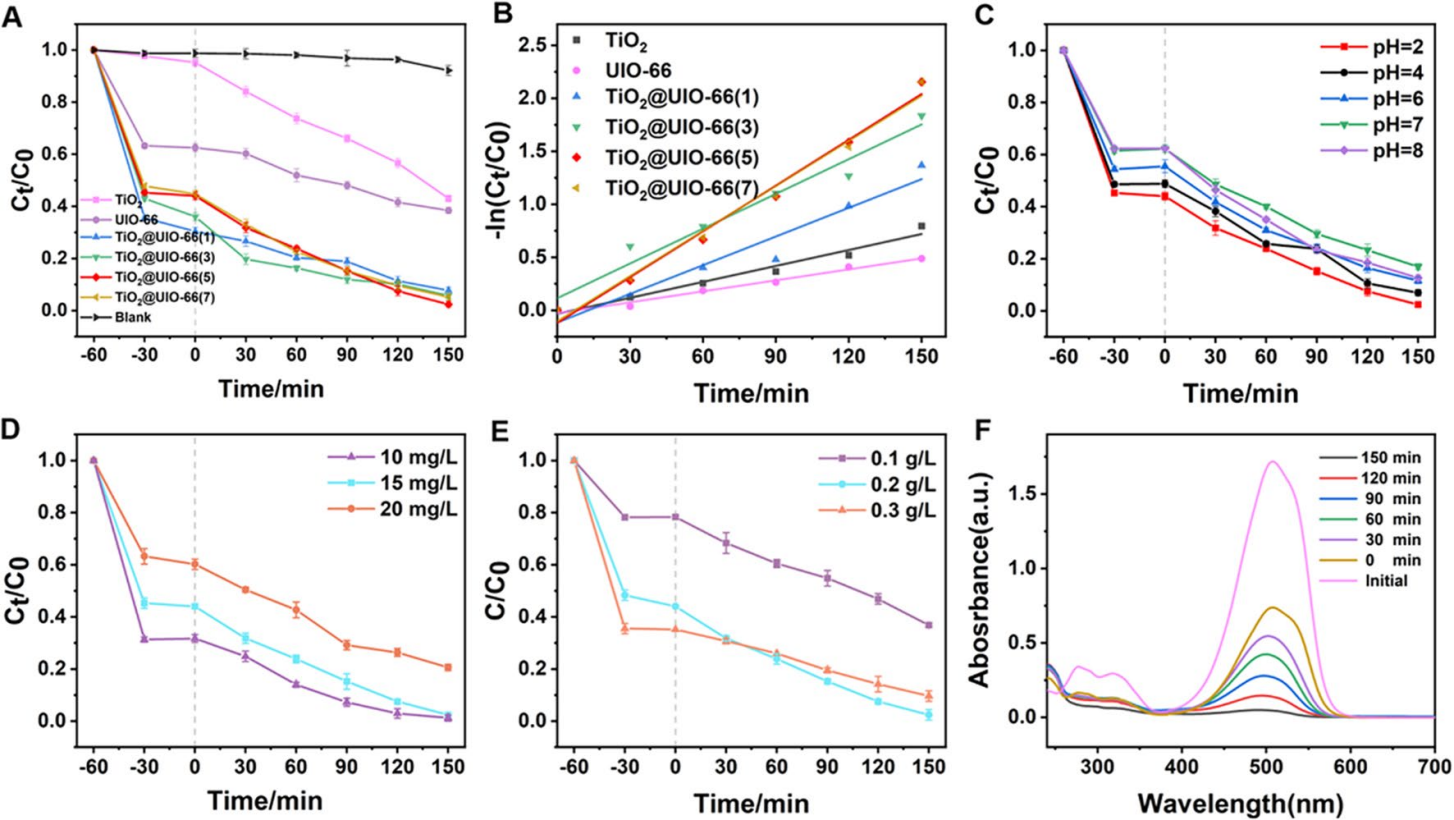
The photocatalytic performance of TiO_2_@UiO-66. **A** Photocatalytic degradation of MO with the composite materials; **B** The fitting curve by a quasi-first-order reaction; **C** The effect of initial pH; **D** MO concentration; **E** Dosage of catalyst; **F** The UV–VIS absorption spectra of MO after photocatalytic degradation

Figure 5A shows the effect of TiO_2_ contents to MO degradation when the photocatalyst dosage is 0.2 g·L^−1^, the concentration of MO is 15 mg·L^−1^ and pH = 2. TiO_2_@UiO-66 composites with different contents of TiO_2_ were prepared with loadings of 1, 3, 5, and 7%, respectively. In Fig. 5A, the adsorption effect of TiO_2_ on MO is very low in the dark reaction stage, while the adsorption ability of UiO-66 on MO is very strong. In the photocatalytic stage, the catalytic capacity of TiO_2_ is much greater than UiO-66(according to the slope of the lines). The effect of adsorption and degradation efficiency of pure TiO_2_ is 56.98% and UiO-66 is 61.58% within 150 min under UV light. In the absence of catalyst, less than 5% of MO was degradation after 150 min. From Fig. 5A, it is quite clear that the degradation efficiency of TiO_2_@UiO-66(x) to MO can reach over 92%, much higher than pure UiO-66 and TiO_2_. The addition of micro-TiO_2_ can greatly improve the degradation efficiency of MO. The sorption of the composites to MO is bigger than that of UiO-66 and TiO_2_ in the dark reaction stage and the degradation efficiency of composites is higher than that of UiO-66 and TiO_2_ in the photocatalytic stage. MO adsorbed on TiO_2_@UiO-66(x) is easy to be degraded because of its close proximity.

The photocatalytic degradation of MO can be analyzed by a quasi-first-order reaction, and their fitting diagram is shown in Fig. 5B. Table S2 lists the kinetic rate of TiO_2_@UiO-66 composite. It can be seen that the kinetic rate of TiO_2_@UiO-66(5) is the highest, which is 0.01438 min^−1^. TiO_2_@UiO-66(5) with a loading of 5% was used in subsequent experiments.

#### Effects of pH

Figure 5C show the effect of pH on MO degradation when the photocatalyst dosage is 0.2 g·L^−1^ and the concentration of MO is 15 mg·L^−1^. It is clear that the TiO_2_@UiO-66(5) composite exhibits excellent photocatalytic activity at pH = 2, with a degradation efficiency of 97.59%. After dark adsorption for 60 min, the adsorption capacities of the solution at pH range 2–8 were 43.04, 47.78, 54.48, 37.76 and 37.63%, respectively. Zeta potential (Fig. S2) shows that the surface charge of the catalyst decreases with increasing pH in the pH range of 2–8, so that TiO_2_@UiO-66(5) composites are more sensitive to the anionic dye MO at low pH. In addition, H^+^ is easily adsorbed on the surface of TiO_2_ at low pH, which makes the TiO_2_ particles positively charged [52, 53]. The positively charged TiO_2_ particles facilitate the transfer of photoinduced electrons, which react with adsorbed O_2_ to generate ·O_2_^−^ (e- + O_2_ → ·O_2_^−^) [29]. The positively charged TiO_2_ particles can also inhibit the recombination of electrons and holes, and generate more ·OH through the reaction of holes with water, thereby enhancing the photocatalytic efficiency [54, 55].

#### Effects of initial concentrations

Figure 5D shows the effects of MO concentration on degradation when the photocatalyst dosage is 0.2 g·L^−1^ and pH = 2. It can be seen that the degradation efficiency of MO depends on its initial concentration, and the photodegradation efficiency reaches to 79.40% when the dye concentration is 20 mg·L^−1^. The increase in the concentration of MO leads to an increase in the amount of dye adsorbed on the catalyst surface, which promotes the enhancement of the degradation efficiency and the amount of the total degradation amount.

#### Effects of photocatalyst dosage

Figure 5E shows the effect of photocatalyst dosage on MO degradation when the concentration of MO is 15 mg·L^−1^ and pH = 2. When the catalyst concentration was 0.2 g·L^−1^, it had the highest degradation efficiency. This can be thought that the increase in the number of photogenerated carriers and the total active surface area with the increase of catalyst dosage, thus the photocatalytic efficiency increases. When the catalyst is added too much, the turbidity of the suspension increases, and the penetration of light decreases, resulting in a decrease in the utilization rate of light. According to previous research reports, the photocatalytic degradation of other organic pollutants also showed a dependence on catalyst dosage [51, 56, 57].

Figure 5F shows the UV–Vis absorption spectra of MO after photocatalytic degradation at a catalyst concentration of 0.2 g·L^−1^, MO concentration at 15 mg·L^−1^, and pH = 2. The characteristic absorption peak intensity of MO at 504 nm decreased significantly when the illumination time increased to 150 min. The results showed that MO was effectively degraded and no other substances were formed.

The photocatalytic activity of TiO_2_@UiO-66(5) for MO is compared with the reported TiO_2_-based catalysts. The data in Table 1 show that the photocatalyst reported in this study is more effective for the photodegradation of MO compared to the catalysts already reported.

**Table 1:** Comparison of the performance of TiO_2_-based materials for photocatalytic degradation of MO

Catalyst	Catalyst amount (g/L)	MO (mg/L)	Light source	efficiency (%)	Ref
TiO_2_	4.00	15	UV light	100.00	[12]
1.5wt.%Pt-TiO_2_/zeolite	3.00	20	UV light	86.20	[58]
CuP-TiO_2_	0.30	20	UV light	98.00	[59]
Ag/TiO_2_	1.00	16	UV light	98.90	[60]
T/M-3%	1.00	20	UV light	99.60	[61]
TiO_2_ NPs	1.25	15	UV light	85.90	[62]
ZnCl_2_/TiO_2_ = 0.2%	1.00	10	UV light	51.00	[63]
NiSO_4_/TiO_2_ = 0.2%	1.00	10	UV light	25.00	[64]
ZnO-TiO_2_/SO_4_^2−^	0.80	20	UV light	90.34	[65]
TiO_2_/Al_2_O_3_	80.00	30	UV light	97.50	[66]
AT_2_-10	2.00	20	UV light	72.79	[67]
Ag/MoO_3_/TiO_2_	0.96	10	UV light	95.60	[68]
TiO_2_/CDs	1.00	20	UV light	98.00	[69]
TiO_2_-Mo (3 wt%)	10.00	5	UV light	97.80	[70]
TQDs/CC	2.00	10	UV light	~ 100.00	[71]
TiO_2_ NPs	1.00	15	UV light	98.00	[72]
TiO_2_/Bentonite/ZnO	4.00	20	UV light	95.00	[73]
black TiO_2_	1.00	10	UV light	82.17	[74]
Ag-TiO_2_	0.25	10	UV light	87.50	[75]
Bi_2_WO_6_/LM-TiO_2_	1.00	20	UV light	93.60	[13]
CFA/TiO_2_	1.00	20	UV light	98.00	[76]
TiO_2_/AC-400	0.80	4	UV light	80.00	[77]
TiO_2_@UiO-66(5)	0.20	15	UV light	97.59	This work

### Cycling performance

To explore the recyclability of the material, TiO_2_@UiO-66(5) composite was used repeatedly three times. Figure S3A shows that the degradation efficiency of TiO_2_@UiO-66(5) composites to MO respectively were 92.54%, 88.76%, and 86.90% after three-cycle experiments. Obviously, the degradation efficiency did not decrease significantly with the increase of the number of cycles. Perhaps the pores of TiO_2_@UiO-66(5) are blocked to some extent when the adsorption of MO in each photocatalytic process and affect the photocatalytic effect. Figure S3B and S3C is the XRD pattern and SEM of TiO_2_@UiO-66(5) after three experiments. Before and after photocatalysis, the crystal structure and morphology of TiO_2_@UiO-66(5) did not change significantly, indicating that the material has good stability and recyclability.

### Water stability and structural stability

The water stability and structural stability of TiO_2_@UiO-66 was evaluated by XRD after being exposed to liquid water for up to 15 days. Figure S4 shows that TiO_2_@UiO-66(5) can preserve the crystal structure perfectly throughout the whole water stability experiment because its XRD patterns remain nearly unchanged for up to 15 days. Our measurements show that the compound shows remarkable water stability and structural stability.

In addition, the TiO_2_@UiO-66(5) after degradation of MO was characterized by SEM and EDS to further investigate its properties. SEM images (Fig. S5) showed that after degradation of MO, TiO_2_@UiO-66(5) remained intact with its original morphology. The composite exhibits an octahedral shape with a large number of TiO_2_ particles coated on the surface. Mapping and EDS (Fig. S5D and Fig. S6) observed that after degradation of MO, the characteristic element in MO, S, appeared on the surface of TiO_2_@UiO-66(5). It indicates that MO indeed reacted on the surface of TiO_2_@UiO-66(5) to complete the degradation.

### Mechanisms for MO photocatalytic degradation

**Fig. 6:**
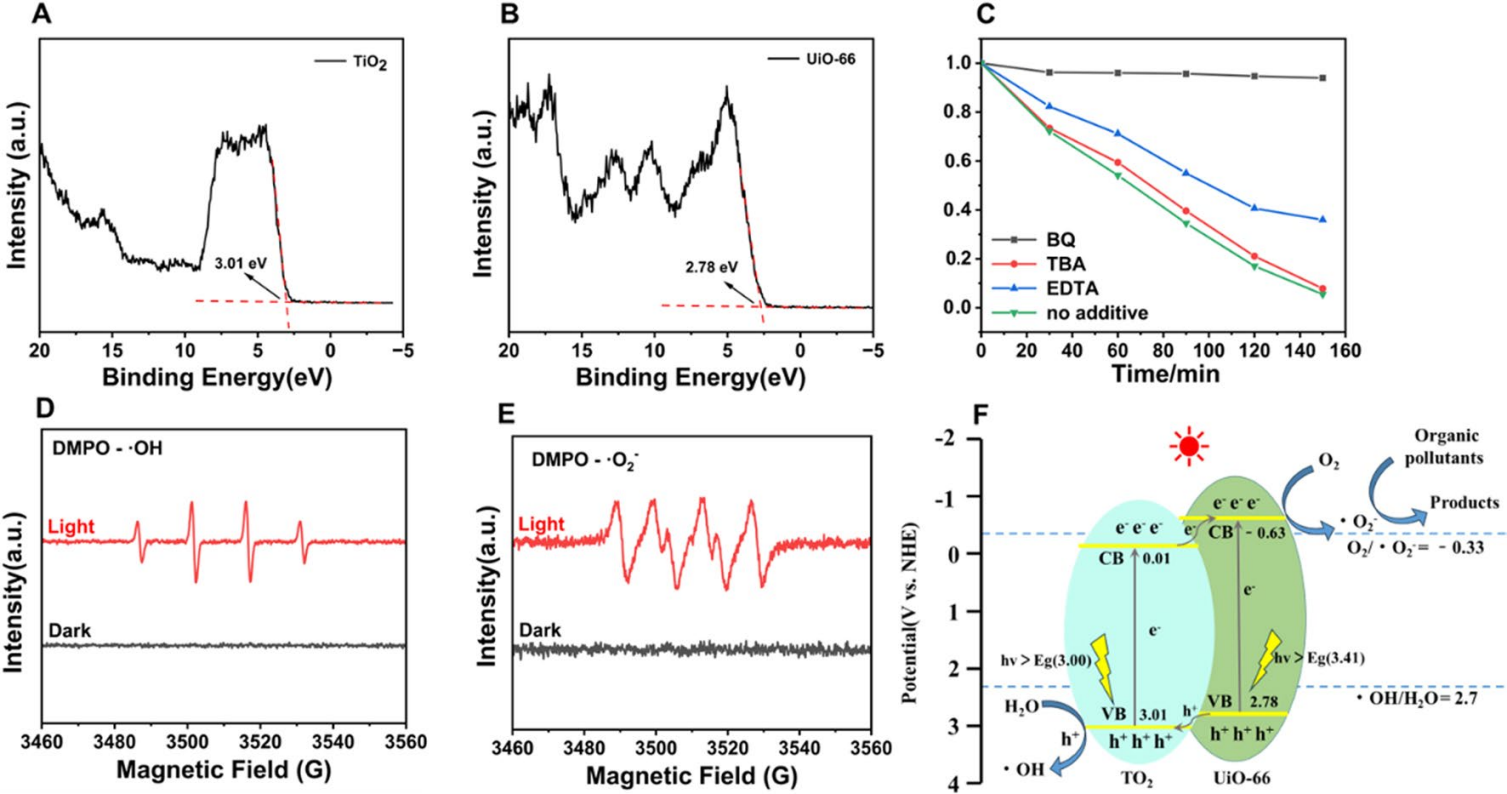
Mechanistic study on the photocatalytic degradation of TiO_2_@UiO-66(5). VB-XPS spectra of **A** TiO_2_ and **B** UiO-66; **C** Effects of Different radical scavengers on photocatalytic efficiency; **D** DMPO-•OH spin-trapping ESR spectra of aqueous TiO_2_@UiO-66(5) dispersion (6 mg/mL) under 250W high-pressure mercury lamp irradiation; **E** DMPO-•O_2_^−^ spin-trapping ESR spectra of TiO_2_@UiO-66(5) dispersed in methanol (6 mg/mL) under 250 W high-pressure mercury lamp irradiation; **F** The proposed electron–hole transfer mechanism at the TiO_2_@UiO-66 interface

From the VB-XPS spectra (Fig. 6A and B), the valence band (VB) values of TiO_2_ and UiO-66 are 3.01 and 2.78 eV, respectively. Energy band theory tells us that we can calculate the values of the conduction band (CB) of TiO_2_ and UiO-66 according to the formula $\mathrm{CB}=\mathrm{VB}-\mathrm{Eg}$, which are 0.01 and − 0.63 eV, respectively. From this, the ΔE_V_ and ΔE_C_ offsets between UiO-66 and TiO_2_ can be calculated to be 0.23 and 0.64 eV, respectively.

Both ΔEc and ΔEv are positive, so it comes out that TiO_2_@UiO-66 is a type-II heterojunction. Since the light intensity and the applied electric field strongly affect the properties of the type II heterojunction, it makes the type-II heterojunction exhibit unusual dynamics of the carriers compared to the type-I heterojunction, which can promote the efficient separation of the photogenerated charge carriers.

To explore the main active species in the degradation process, isopropanol (TBA), EDTA-2Na, and benzoquinone (BQ) at the same concentrations were used to quench OH radical, hole, and ·O_2_^−^ radical, respectively. In the presence of BQ, the effect of photocatalytic degradation of MO decreased significantly, indicating that ·O_2_^−^ plays a major role in photocatalytic processes (Fig. 6C). When TBA is added in the solution, the catalytic activity of TiO_2_@UiO-66(5) is hardly different with that of without TBA. It can be considered that ·OH is not the main active species of MO degradation. The amount of ·O_2_^−^ decreases with the electrons decrease when the holes are consumed by EDTA-2Na. Therefore, the catalytic efficiency of MO degradation decreases. In summary, TiO_2_@UiO-66 provides ·O_2_^−^ which reacts with organic matter to produce degradation products and water, while ·OH is only an auxiliary catalytic group.

To further confirm that there is radical generation in the photocatalytic system, we used ESR spectroscopic technology. As shown in Fig. 6D and E, the characteristic signal of DMPO-•OH and DMPO-•O_2_- was not observed under dark conditions. After 5 min of irradiation with a high-pressure mercury lamp, we observed the characteristic signals of DMPO-•OH and DMPO-•O_2_-. The results showed that •OH- and •O_2_- radicals were indeed generated in this photocatalytic system, which is consistent with the previous experimental results.

Based on the above discussion, we propose a possible mechanism for TiO_2_@UiO-66 photocatalytic degradation of MO(Fig. 6F). According to the photodegradation principle, the ground state electrons on the VB can excite the CB after absorbing photons, making CB carry free electrons and leaving holes on the VB. The oxidation reaction occurs when the VB potential of the photocatalyst is higher than the redox potential of the ·OH radical (E_0_(H_2_O/·OH) = 2.7 V vs. NHE). The reduction reaction occurs when the CB potential of the photocatalyst is lower than the redox potential of the ·O_2_^−^ radical (E_0_(O_2_/·O_2_^−^) = -0.28 V vs. NHE) [78]. Holes on the VB are strong oxidants and electrons on CB are strong reducing agents. The holes react with H_2_O to produce chemically active free radical groups (·OH) (Eq. 1), the combination of electrons on the CB and O_2_ also produces chemically active free radical groups (·O_2_^−^) (Eq. 2). ·OH and·O_2_^−^ radicals degrade with organic contaminants (Eqs. 3, 4). Under illumination, UiO-66 and TiO_2_ in TiO_2_@UiO-66 composites can be excited by ultraviolet light, generating electrons and holes on CB and VB, respectively. Both potentials of UiO-66 and TiO_2_ are higher than H_2_O/·OH (2.7 eV vs. NHE), so some of the h + generates ·OH on the material surface combining with H_2_O. Meanwhile, the CB potential of UiO-66 is lower than O_2_/·O_2_^−^ (-0.3 eV vs. NHE), so the e- on UiO-66 is easily trapped by oxygen generating ·O_2_^−^ (Fig. 6F) [79]. Due to the tight combination of UiO-66 and TiO_2_, the electrons on the CB of TiO_2_ are transferred to the CB of UiO-66. The quantity of ·O_2_^−^ produced by the composite catalyst is larger than that of the single catalyst.

Based on the obtained results, the enhanced photoactivity of the composite is mainly attributed to a decrease in Eg and an improved electron–hole separation.

1 $$\begin{array}{c}{\mathrm{H}}_{2}O+{\mathrm{h}}_{\mathrm{vb}}^{+}\to \bullet OH+{\mathrm{H}}^{+}\#\end{array}$$

2 $$\begin{array}{c}{\mathrm{O}}_{2}+{\mathrm{e}}_{\mathrm{cb}}^{-}\to \bullet {\mathrm{O}}_{2}^{-}\#\end{array}$$

3 $$\begin{array}{c}Dye+\bullet OH\to {\mathrm{H}}_{2}O+dye intermediates\#\end{array}$$

4 $$\begin{array}{c}Dye+\bullet {\mathrm{O}}_{2}^{-}\to {\mathrm{H}}_{2}O+dye intermediates\#\end{array}$$

## Conclusions

The photocatalytic degradation of MO by TiO_2_ loaded with UiO-66 was investigated for the first time. The composite MOFs catalyst, TiO_2_@UiO-66 was prepared by adding micro-TiO_2_ by solvothermal method, and their photocatalytic performance was explored with MO as the target pollutant. In the study of influencing conditions, the effects of TiO_2_ loading, solution pH, catalyst dosage, and initial solution concentration on the photocatalytic degradation were investigated. The photocatalytic activity of the TiO_2_@UiO-66 composite was higher than that of TiO_2_ and UiO-66, and its degradation rate of MO could reach 97.59%. After three cycles, the degradation rate of MO still reaches 86.90%, showing excellent photocatalytic activity and good cycling performance. Its excellent photocatalytic performance can be attributed to two aspects: (1) The huge specific surface area of UiO-66 can significantly improve the adsorption effect of TiO_2_. (2) The close contact interface between TiO_2_ and UiO-66 can effectively separate and transfer photogenerated carriers. The free radical trapping experiments show that ·O_2_^−^ is the active species that play a major role in the degradation. Moreover, TiO_2_@UiO-66 still has an intact structure after being immersed in aqueous solution for 15 days, and it has excellent water stability. The results demonstrated that TiO_2_@UiO-66 is a kind of catalyst with simple preparation, stable structure and good catalytic performance for the purification of environmental organic contaminants.

### Supplementary Information

**Additional file 1**. Supplementary Material.

## Data Availability

All data generated or analysed during this study are included in this published article and its supplementary information files.

## References

[1] Daghrir R, Drogui P, Robert D (2013). Modified TiO_2_ for environmental photocatalytic applications: a review. Ind Eng Chem Res.

[2] Tian C, Zhang Q, Wu A, Jiang M, Liang Z, Jiang B, Fu H (2012). Cost-effective large-scale synthesis of ZnO photocatalyst with excellent performance for dye photodegradation. Chem Commun.

[3] Lin YF, Chen HW, Chien PS, Chiou CS, Liu CC (2011). Application of bifunctional magnetic adsorbent to adsorb metal cations and anionic dyes in aqueous solution. J Membr Sci.

[4] Zhu MX, Lee L, Wang HH, Wang Z (2007). Removal of an anionic dye by adsorption/precipitation processes using alkaline white mud. J Hazard Mater.

[5] Li W, Mu B, Yang Y (2019). Feasibility of industrial-scale treatment of dye wastewater via bio-adsorption technology. Bioresour Technol.

[6] Wu R, Wang S, Zhou Y, Long J, Dong F, Zhang W (2019). Chromium-based metal–organic framework MIL-101 decorated with CdS quantum dots for the photocatalytic synthesis of imines. ACS Appl Nano Mater.

[7] Wang L, Li Z, Chen J, Huang Y, Zhang H, Qiu H (2019). Enhanced photocatalytic degradation of methyl orange by porous graphene/ZnO nanocomposite. Environ Pollut.

[8] Li X, Kang B, Dong F, Zhang Z, Luo X, Han L, Huang J, Feng Z, Chen Z, Xu J, Peng B, Wang ZL (2021). Enhanced photocatalytic degradation and H_2_/H_2_O_2_ production performance of S-pCN/WO272 S-scheme heterojunction with appropriate surface oxygen vacancies. Nano Energy.

[9] Li X, Luo Q, Han L, Deng F, Yang Y, Dong F (2022). Enhanced photocatalytic degradation and H_2_ evolution performance of N CDs/S-C_3_N_4_ S-scheme heterojunction constructed by π-π conjugate self-assembly. J Mater Sci Technol.

[10] Li X, Xiong J, Gao X, Ma J, Chen Z, Kang B, Liu J, Li H, Feng Z, Huang J (2020). Novel BP/BiOBr S-scheme nano-heterojunction for enhanced visible-light photocatalytic tetracycline removal and oxygen evolution activity. J Hazard Mater.

[11] Xiong J, Li X, Huang J, Gao X, Chen Z, Liu J, Li H, Kang B, Yao W, Zhu Y (2020). CN/rGO@BPQDs high-low junctions with stretching spatial charge separation ability for photocatalytic degradation and H_2_O_2_ production. Appl Catal B Environ.

[12] Al-Qaradawi S, Salman RS (2002). Photocatalytic degradation of methyl orange as a model compound. J Photoch Photobio A.

[13] Zhang L-Y, Yang J-J, Han Y-L (2022). Novel adsorption-photocatalysis integrated bismuth tungstate modified layered mesoporous titanium dioxide (Bi_2_WO_6_/LM-TiO_2_) composites. Opt Mater.

[14] Ahmadijokani F, Molavi H, Rezakazemi M, Tajahmadi S, Bahi A, Ko F, Aminabhavi TM, Li J-R, Arjmand M (2022). UiO-66 metal–organic frameworks in water treatment: A critical review. Prog Mater Sci.

[15] Ahmadijokani F, Molavi H, Tajahmadi S, Rezakazemi M, Amini M, Kamkar M, Rojas OJ, Arjmand M (2022). Coordination chemistry of metal–organic frameworks: Detection, adsorption, and photodegradation of tetracycline antibiotics and beyond. Coord Chem Rev.

[16] Elkin T, Saouma CT (2019). Effect of linker and metal on photoreduction and cascade reactions of nitroaromatics by M-UiO-66 metal organic frameworks. Inorg Chim Acta.

[17] Vermoortele F, Vandichel M, Van de Voorde B, Ameloot R, Waroquier M, Van Speybroeck V, De Vos DE (2012). Electronic effects of linker substitution on Lewis acid catalysis with metal-organic frameworks. Angew Chem Int Ed Engl.

[18] Choi S, Lee HE, Ryu CH, Lee J, Lee J, Yoon M, Kim Y, Park MH, Lee KM, Kim M (2019). Synthesis of o-carborane-functionalized metal-organic frameworks through ligand exchanges for aggregation-induced emission in the solid state. Chem Commun.

[19] Chen S-Z, Zhang P-Y, Zhu W-P, Chen L, Xu S-M (2006). Deactivation of TiO_2_ photocatalytic films loaded on aluminium: XPS and AFM analyses. Appl Surf Sci.

[20] Yu B, Meng F, Khan MW, Qin R, Liu X (2020). Synthesis of hollow TiO_2_@g-C_3_N_4_/Co_3_O_4_ core-shell microspheres for effective photooxidation degradation of tetracycline and MO. Ceram Int.

[21] Zhang J, Guo Z, Yang Z, Wang J, Xie J, Fu M, Hu Y (2020). TiO_2_@UiO-66 composites with efficient adsorption and photocatalytic oxidation of VOCs: investigation of synergistic effects and reaction mechanism. ChemCatChem.

[22] Alotaibi AM, Promdet P, Hwang GB, Li J, Nair SP, Sathasivam S, Kafizas A, Carmalt CJ, Parkin IP (2021). Zn and N codoped TiO_2_ thin films: photocatalytic and bactericidal activity. ACS Appl Mater Interfaces.

[23] Wang F, Ma Z, Ban P, Xu X (2017). C, N and S codoped rutile TiO_2_ nanorods for enhanced visible-light photocatalytic activity. Mater Lett.

[24] Ge H, Xu F, Cheng B, Yu J, Ho W (2019). S-Scheme heterojunction TiO_2_/CdS nanocomposite nanofiber as H_2_-production photocatalyst. ChemCatChem.

[25] Wang Y, Liu H, Zhang M, Duan W, Liu B (2017). A dual-functional UiO-66/TiO_2_ composite for water treatment and CO_2_ capture. RSC Adv.

[26] Man Z, Meng Y, Lin X, Dai X, Wang L, Liu D (2022). Assembling UiO-66@TiO_2_ nanocomposites for efficient photocatalytic degradation of dimethyl sulfide. Chem Eng J.

[27] Ma Y, Tang Q, Sun W-Y, Yao Z-Y, Zhu W, Li T, Wang J (2020). Assembling ultrafine TiO_2_ nanoparticles on UiO-66 octahedrons to promote selective photocatalytic conversion of CO_2_ to CH_4_ at a low concentration. Appl Catal B Environ.

[28] Zhang J, Hu Y, Qin J, Yang Z, Fu M (2020). TiO_2_-UiO-66-NH_2_ nanocomposites as efficient photocatalysts for the oxidation of VOCs. Chem Eng J.

[29] Li YX, Wang X, Wang CC, Fu H, Liu Y, Wang P, Zhao C (2020). S-TiO_2_/UiO-66-NH_2_ composite for boosted photocatalytic Cr(VI) reduction and bisphenol A degradation under LED visible light. J Hazard Mater.

[30] Sun T, Zhang X, Hu Y, Xu L, Zhao Y (2022). Design and enhancement of photocatalytic activity of porphyrin functionalized UiO-66 and Keggin unit co-doped titanium dioxide heterojunction. Appl Surf Sci.

[31] Ling L, Wang Y, Zhang W, Ge Z, Duan W, Liu B (2018). Preparation of a novel ternary composite of TiO_2_/UiO-66-NH_2_/graphene oxide with enhanced photocatalytic activities. Catal Letters.

[32] Zhu G, Feng S, Chao J, Zheng W, Shao C (2020). One-pot synthesis of C-dots modified TiO_2_ nanosheets/UiO-66-NH_2_ with improved photocatalytic activity under visible light. Ceram Int.

[33] Liu B, Liu X, Liu J, Feng C, Li Z, Li C, Gong Y, Pan L, Xu S, Sun CQ (2018). Efficient charge separation between UiO-66 and ZnIn_2_S_4_ flowerlike 3D microspheres for photoelectronchemical properties. Appl Catal B Environ.

[34] Zhang R, Du B, Li Q, Cao Z, Feng G, Wang X (2019). α-Fe_2_O_3_ nanoclusters confined into UiO-66 for efficient visible-light photodegradation performance. Appl Surf Sci.

[35] Zhang X, Zhang N, Gan C, Liu Y, Chen L, Zhang C, Fang Y (2019). Synthesis of In2S3/UiO-66 hybrid with enhanced photocatalytic activity towards methyl orange and tetracycline hydrochloride degradation under visible-light irradiation. Mat Sci Semicon Proc.

[36] Zhuang H, Chen B, Cai W, Xi Y, Ye T, Wang C, Lin X (2019). UiO-66-supported Fe catalyst: a vapour deposition preparation method and its superior catalytic performance for removal of organic pollutants in water. Roy Soc Open Sci.

[37] Li S, Wang X, He Q, Chen Q, Xu Y, Yang H, Lü M, Wei F, Liu X (2016). Synergistic effects in N-K_2_Ti_4_O_9_/UiO-66-NH_2_ composites and their photocatalysis degradation of cationic dyes. Chinese J Catal.

[38] Deng Q, Zhang W, Lan T, Xie J, Xie W, Liu Z, Huang Y, Wei M (2018). Anatase TiO2 quantum dots with a narrow band gap of 2.85 eV based on surface hydroxyl groups exhibiting significant photodegradation property. Eur J Inorg Chem.

[39] Shearer GC, Vitillo JG, Bordiga S, Svelle S, Olsbye U, Lillerud KP (2016). Functionalizing the defects: postsynthetic ligand exchange in the metal organic framework UiO-66. Chem Mater.

[40] Schaate A, Roy P, Godt A, Lippke J, Waltz F, Wiebcke M, Behrens P (2011). Modulated synthesis of Zr-based metal-organic frameworks: from nano to single crystals. Chem.

[41] Cavka JH, Jakobsen S, Olsbye U, Guillou N, Lamberti C, Bordiga S, Lillerud KP (2008). A new zirconium inorganic building brick forming metal organic frameworks with exceptional stability. Jacks.

[42] Karimian N, Fakhri H, Amidi S, Hajian A, Arduini F, Bagheri H (2019). A novel sensing layer based on metal–organic framework UiO-66 modified with TiO_2_–graphene oxide: application to rapid, sensitive and simultaneous determination of paraoxon and chlorpyrifos. New J Chem.

[43] Fu YY, Yang CX, Yan XP (2013). Incorporation of metal-organic framework UiO-66 into porous polymer monoliths to enhance the liquid chromatographic separation of small molecules. Chem Commun.

[44] Abdelhamid HN (2020). UiO-66 as a catalyst for hydrogen production via the hydrolysis of sodium borohydride. Dalton Trans.

[45] Zhang X, Yang Y, Lv X, Wang Y, Liu N, Chen D, Cui L (2019). Adsorption/desorption kinetics and breakthrough of gaseous toluene for modified microporous-mesoporous UiO-66 metal organic framework. J Hazard Mater.

[46] Zhao S, Chen D, Xu H, Mei J, Qu Z, Liu P, Cui Y, Yan N (2018). Combined effects of Ag and UiO-66 for removal of elemental mercury from flue gas. Chemosphere.

[47] Bariki R, Majhi D, Das K, Behera A, Mishra BG (2020). Facile synthesis and photocatalytic efficacy of UiO-66/CdIn_2_S_4_ nanocomposites with flowerlike 3D-microspheres towards aqueous phase decontamination of triclosan and H_2_ evolution. Appl Catal B Environ.

[48] Shi J, Zhang L, Xiao P, Huang Y, Chen P, Wang X, Gu J, Zhang J, Chen T (2018). Biodegradable PLA nonwoven fabric with controllable wettability for efficient water purification and photocatalysis degradation. ACS Sustain Chem Eng.

[49] Lin Y, Yang C, Wu S, Li X, Chen Y, Yang WL (2020). Construction of built-In electric field within silver phosphate photocatalyst for enhanced removal of recalcitrant organic pollutants. Adv Func Mater.

[50] An R, Zhang F, Zou X, Tang Y, Liang M, Oshchapovskyy I, Liu Y, Honarfar A, Zhong Y, Li C, Geng H, Chen J, Canton SE, Pullerits T, Zheng K (2018). Photostability and photodegradation processes in colloidal CsPbI3 perovskite quantum dots. ACS Appl Mater Interfaces.

[51] Krishnan A, Vishwanathan PV, Mohan AC, Panchami R, Viswanath S, Krishnan AV (2021). Tuning of photocatalytic performance of CeO_2_-Fe_2_O_3_ composite by Sn-doping for the effective degradation of methlene blue (MB) and methyl orange (MO) dyes. Surf Interfaces.

[52] Li ZJ, Huang ZW, Guo WL, Wang L, Zheng LR, Chai ZF, Shi WQ (2017). Enhanced photocatalytic removal of Uranium(VI) from aqueous solution by magnetic TiO_2_/Fe_3_O_4_ and its graphene composite. Environ Sci Technol.

[53] Li P, Wang J, Wang Y, Liang J, He B, Pan D, Fan Q, Wang X (2019). Photoconversion of U(VI) by TiO_2_: an efficient strategy for seawater uranium extraction. Chem Eng J.

[54] Huang Q, Hu Y, Pei Y, Zhang J, Fu M (2019). In situ synthesis of TiO_2_@NH_2_-MIL-125 composites for use in combined adsorption and photocatalytic degradation of formaldehyde. Appl Catal B Environ.

[55] Zhang Y, Wan J, Ke Y (2010). A novel approach of preparing TiO_2_ films at low temperature and its application in photocatalytic degradation of methyl orange. J Hazard Mater.

[56] Chang Chien S-W, Ng D-Q, Kumar D, Lam S-M, Jaffari ZH (2022). Investigating the effects of various synthesis routes on morphological, optical, photoelectrochemical and photocatalytic properties of single-phase perovskite BiFeO_3_. J Phys Chem Solids.

[57] Krishnan A, Viswanath S, Mohan AC, Panchami R, Vishwanathan PV (2021). Surface engineering of Ni-P electrode by cobalt oxide co-deposition for electrochemical hydrogen evolution reaction. J Environ Chem Eng.

[58] Huang M, Xu C, Wu Z, Huang Y, Lin J, Wu J (2008). Photocatalytic discolorization of methyl orange solution by Pt modified TiO_2_ loaded on natural zeolite. Dyes Pigm.

[59] Zhou XT, Ji HB, Huang XJ (2012). Photocatalytic degradation of methyl orange over metalloporphyrins supported on TiO_2_ Degussa P25. Molecules.

[60] Saravanan R, Manoj D, Qin J, Naushad M, Gracia F, Lee AF, Khan MM, Gracia-Pinilla MA (2018). Mechanothermal synthesis of Ag/TiO_2_ for photocatalytic methyl orange degradation and hydrogen production. Process Saf Environ.

[61] Li H, Sun B, Gao T, Li H, Ren Y, Zhou G (2022). Ti_3_C_2_ MXene co-catalyst assembled with mesoporous TiO_2_ for boosting photocatalytic activity of methyl orange degradation and hydrogen production. Chinese J Catal.

[62] Rashid Al-Mamun M, Hossain KT, Mondal S, Afroza Khatun M, Shahinoor Islam M, Zaved Hossain Khan DM (2022). Synthesis, characterization, and photocatalytic performance of methyl orange in aqueous TiO_2_ suspension under UV and solar light irradiation. S Afr J Chem Eng.

[63] Regraguy B, Ellouzi I, Mabrouki J, Rahmani M, Drhimer F, Mahmou C, Dahchour A, El Mrabet M, El Hajjaji S (2022). Zinc doping of different nanoparticles of TiO_2_ Sachtopore for improved elimination of the methyl orange by photocatalysis. Emerg Mater.

[64] Regraguy B, Rahmani M, Mabrouki J, Drhimer F, Ellouzi I, Mahmou C, Dahchour A, Mrabet ME, Hajjaji SE (2022). Photocatalytic degradation of methyl orange in the presence of nanoparticles NiSO_4_/TiO_2_. Nanotechnol Environ Eng.

[65] You J, Zhang L, He L, Zhang B (2022). Photocatalytic degradation of methyl orange on ZnO–TiO_2_/SO4_2_− heterojunction composites. Opt Mater.

[66] Liu D-J, Lei J-H, Wei S, Jiang B-L, Xie Y-T (2022). Degrading methyl orange via prepare high dispersed TiO_2_/Al_2_O_3_ photocatalyst by combining anodizing and hydro-thermal technology. AIP Adv.

[67] Liu Y, Xiang Y, Xu H, Li H (2022). The reuse of nano-TiO_2_ under different concentration of CO_3_^2–^ using coagulation process and its photocatalytic ability in treatment of methyl orange. Sep Purif Technol.

[68] Kader S, Al-Mamun MR, Suhan MBK, Shuchi SB, Islam MS (2022). Enhanced photodegradation of methyl orange dye under UV irradiation using MoO_3_ and Ag doped TiO_2_ photocatalysts. Environ Technol Innov.

[69] Kuldeep AR, Waghmare RD, Garadkar KM (2022). Green synthesis of TiO_2_/CDs nanohybrid composite as an active photocatalyst for the photodegradation of methyl orange. J Mater Sci-Mater El.

[70] Kanakaraju D, Jasni MAA, Lim YC (2021). A highly photoresponsive and efficient molybdenum-modified titanium dioxide photocatalyst for the degradation of methyl orange. Int J Environ Sci Te.

[71] Shen H, Zhang W, Guo C, Zhu J, Cui J, Xue Z, Chen P (2022). Natural cotton cellulose-supported TiO_2_ quantum dots for the highly efficient photocatalytic degradation of dyes. Nanomater-Basel.

[72] Silva-Osuna ER, Vilchis-Nestor AR, Villarreal-Sanchez RC, Castro-Beltran A, Luque PA (2022). Study of the optical properties of TiO_2_ semiconductor nanoparticles synthesized using Salvia rosmarinus and its effect on photocatalytic activity. Opt Mater.

[73] Dinari A, Mahmoudi J (2022). Response surface methodology analysis of the photodegradation of methyl orange dye using synthesized TiO_2_/Bentonite/ZnO composites. Adv Environ Technol..

[74] Saensook S, Sirisuk A (2022). A factorial experimental design approach to obtain defect-rich black TiO_2_ for photocatalytic dye degradation. J Water Process Eng..

[75] Wu L, Pei X, Mei M, Li Z, Lu S (2022). Study on photocatalytic performance of Ag/TiO_2_ modified cement mortar. Materials.

[76] Liang Y, Chen S, Zhong J, Ding H, Zhu Z, Li S (2022). Acid-etched coal fly ash/TiO_2_ nanocomposites with high photocatalytic degradation efficiency: a high value-added application of coal fly ash. J Sol-Gel Sci Techn.

[77] Arutanti O, Sari AL, Kartikowati CW, Sari AA, Arif AF (2022). Design and application of homogeneous-structured TiO_2_/Activated carbon nanocomposite for adsorption–photocatalytic degradation of MO. Water Air Soil Poll.

[78] Sherly ED, Vijaya JJ, Kennedy LJ, Meenakshisundaram A, Lavanya M (2016). A comparative study of the effects of CuO, NiO, ZrO_2_ and CeO_2_ coupling on the photocatalytic activity and characteristics of ZnO. Korean J Chem Eng.

[79] Li H, Gan S, Wang H, Han D, Niu L (2015). Intercorrelated superhybrid of AgBr supported on graphitic-C_3_N_4_-decorated nitrogen-doped graphene: High engineering photocatalytic activities for water purification and CO_2_ reduction. Adv Mater.

