# Supplementary material for: High performance photocatalyst TiO2@UiO-66 applied to degradation of methyl orange
Source: Discov Nano. 2023 Sep 11;18(1):112. doi: 10.1186/s11671-023-03894-6 (PMC10495301; doi:10.1186/s11671-023-03894-6)
Supplement: Supplementary file 1 — Additional file 1. Supplementary Material. [file 11671_2023_3894_MOESM1_ESM.docx]

**High performance photocatalyst TiO_2_@UiO-66 applied to degradation of methyl orange**

*Jingyi Yang^1†^, Xue Chang^1†^, Fang Wei^1^, Zixiao Lv^1^, Huiling Liu^1^, Zhan Li^1,2*^, Wangsuo Wu^1,3^, Lijuan Qian^1,3*^*

^†^These authors contributed equally to this work.

Supplementary Material


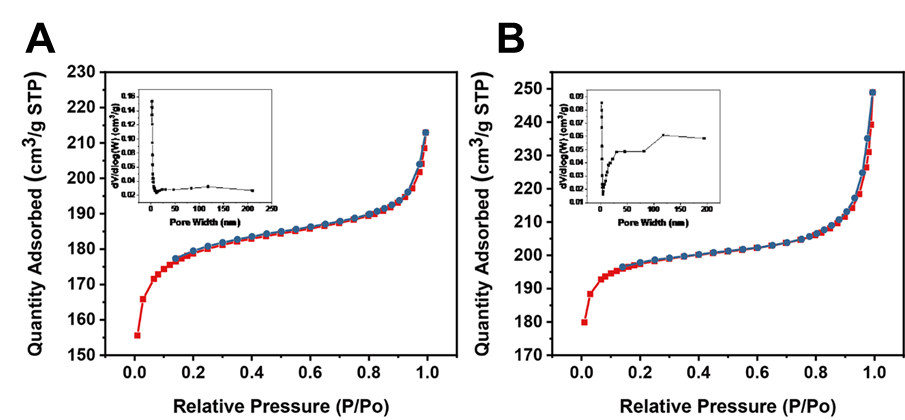


**Fig. S1** (A) Adsorption-desorption isotherms and pore size distributions of UiO-66; (B) Adsorption-desorption isotherms and pore size distributions of TiO_2_@UiO-66.


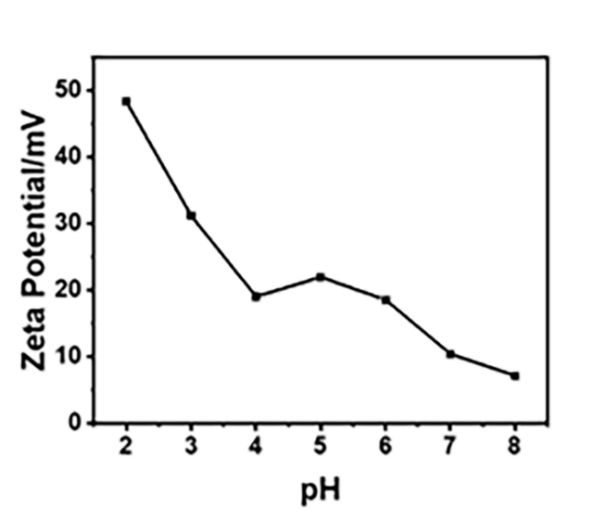


**Fig. S2** Zeta potential of TiO_2_@UiO-66(5) at different pH values.


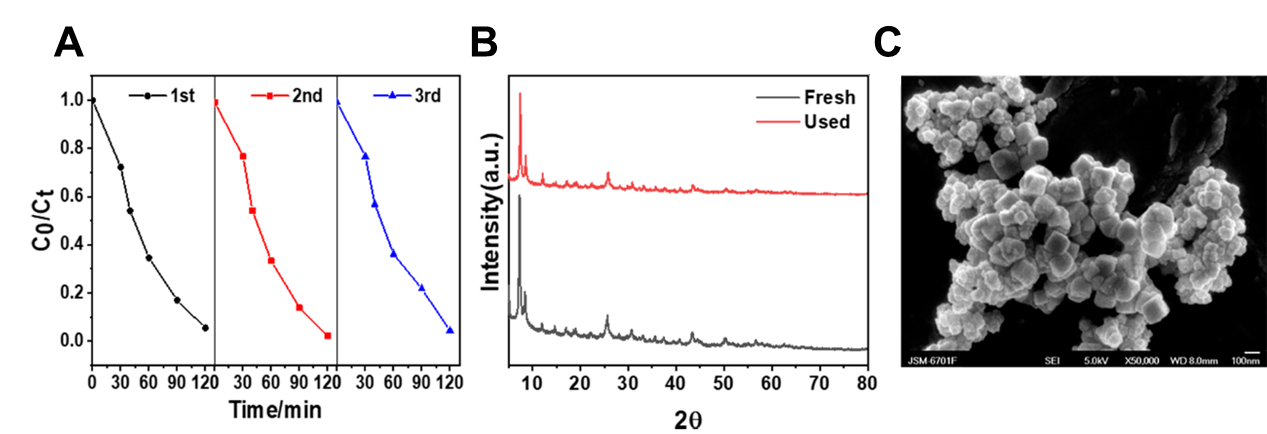


**Fig. S3** (A) Recycling test for MO degradation over TiO_2_@UiO-66(5); (B) XRD pattern of TiO_2_@UiO-66(5) after recycling; (C) SEM diagram of TiO_2_@UiO-66(5) after recycling.


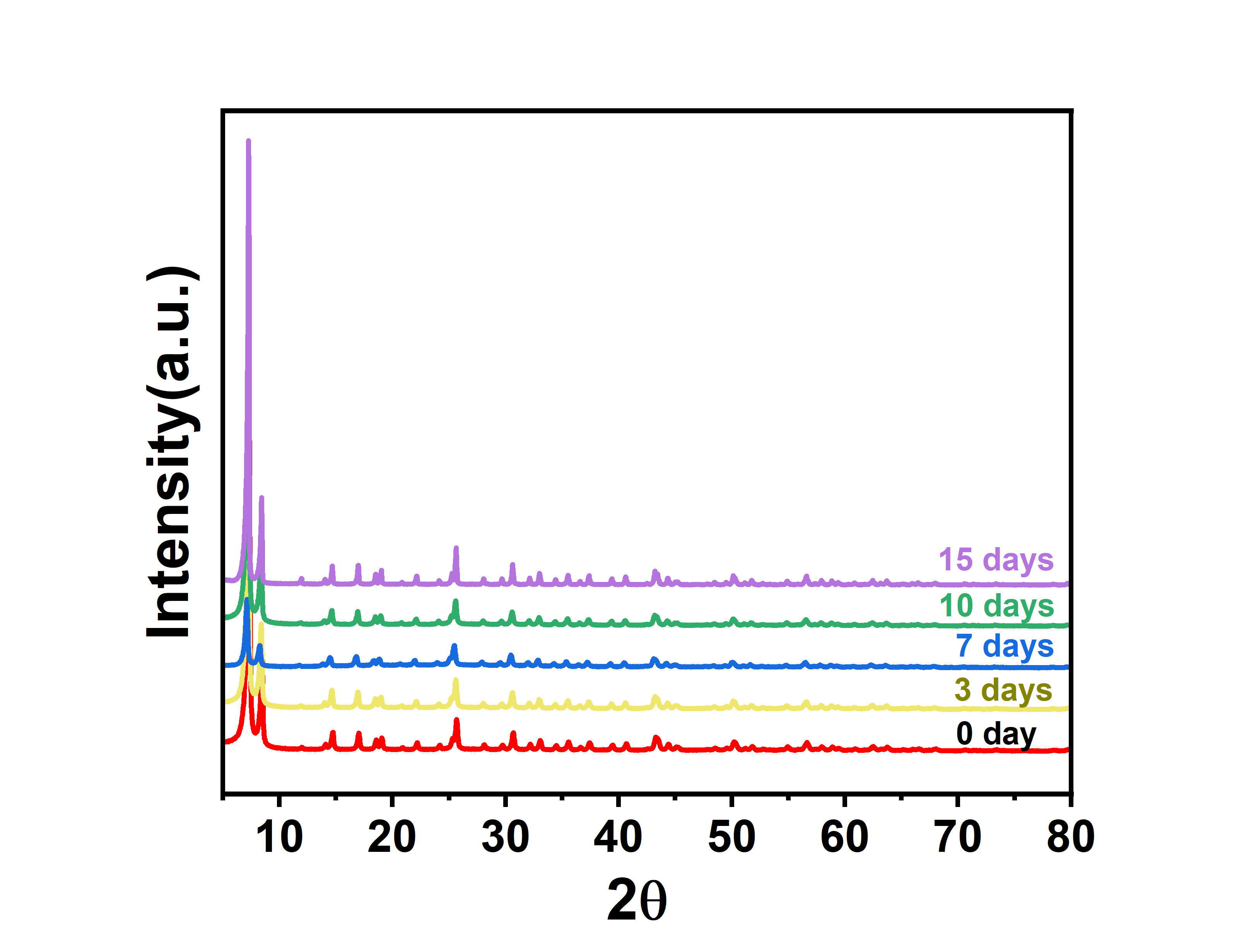


**Fig. S4** XRD patterns of the TiO_2_@UiO-66(5) before and after soaking in liquid water for different periods of time.

Table S1 BET results of UiO-66 and TiO_2_@UiO-66

| **sample** | **BET area(m^2^·g^-1^)** | **Pore width (nm)** |
| --- | --- | --- |
| UiO-66 | 775.12 | 2.45 |
| TiO_2_@UiO-66(5) | 685.94 | 1.98 |

Table S2 Fitting diagram of pseudo-first-order reaction kinetics during the degradation of TiO_2_, UiO-66 and TiO_2_@UiO-66

| **sample** | **k(min^-1^)** | **R^2^** |
| --- | --- | --- |
| TiO_2_@UiO-66(1) | 0.00902 | 0.9418 |
| TiO_2_@UiO-66(3) | 0.01094 | 0.9635 |
| TiO_2_@UiO-66(5) | 0.01438 | 0.9863 |
| TiO_2_@UiO-66(7) | 0.01419 | 0.9860 |


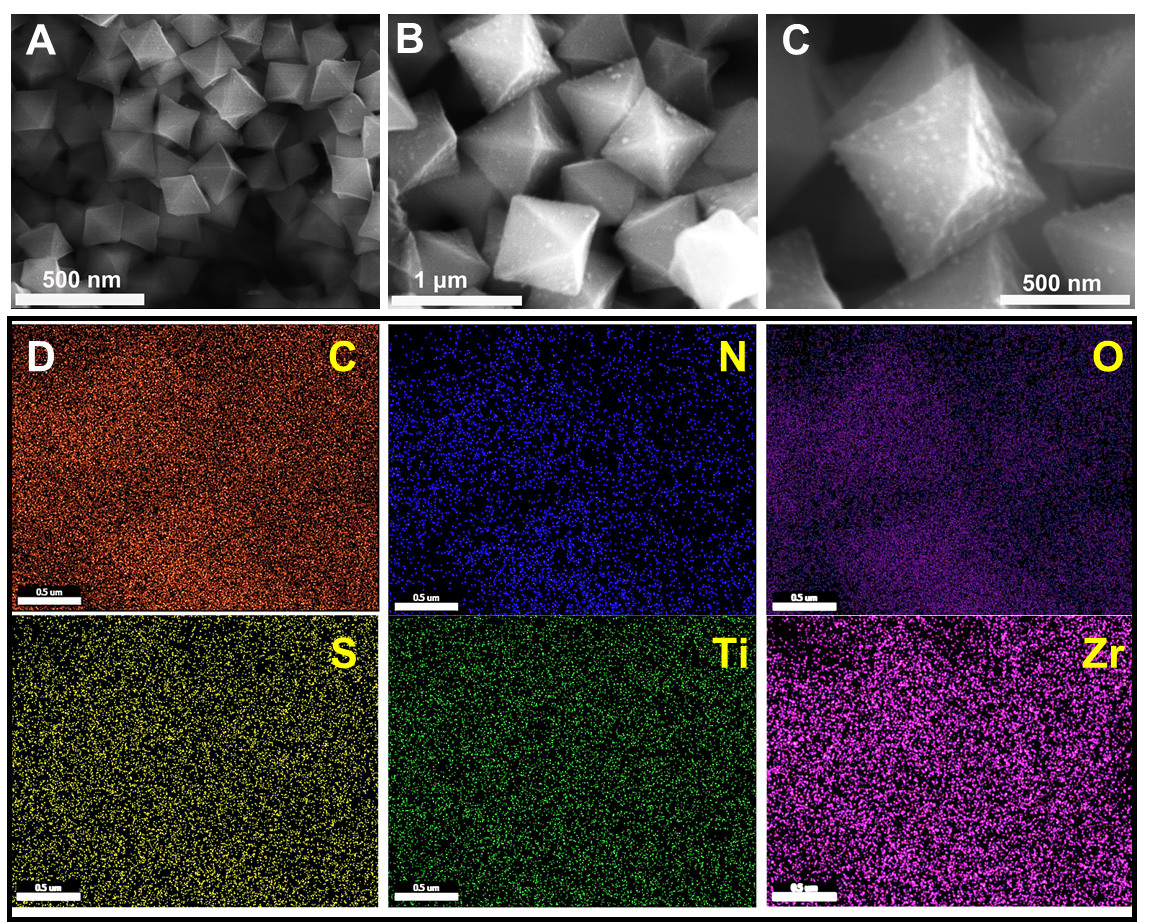


**Fig. S5** SEM and EDS characterization of TiO_2_@UiO-66(5) after degradation of MO. (A-C) SEM images of different sizes; (D) the mapping image of Figure S5C.


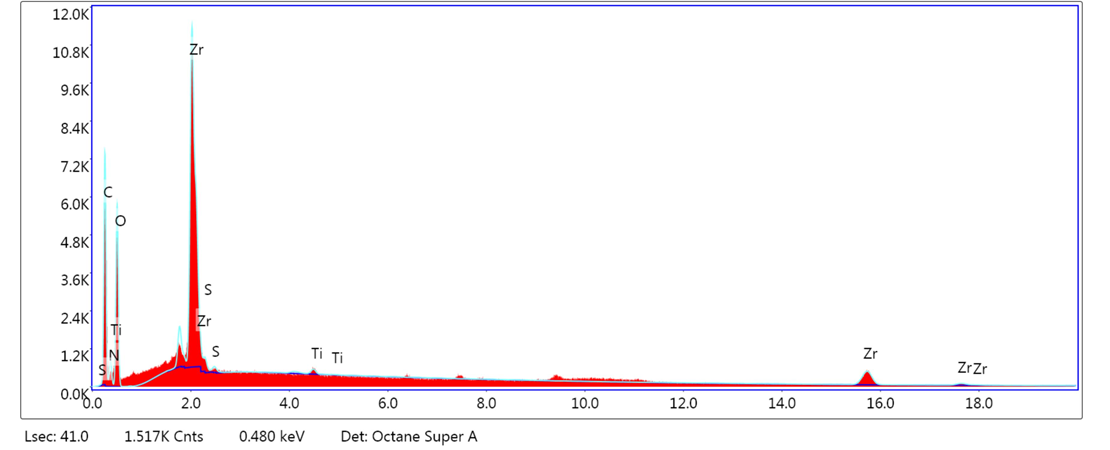


**Fig. S6** EDS analysis spectrum of TiO_2_@UiO-66(5) after degradation of MO.

**Table S3** EDS of TiO_2_@UiO-66(5) after degradation of MO

| Element | Weight % | Atomic % |
| --- | --- | --- |
| C | 41.61 | 55.70 |
| N | 8.23 | 9.45 |
| O | 31.26 | 31.42 |
| S | 0.26 | 0.13 |
| Ti | 0.16 | 0.05 |
| Zr | 18.49 | 3.26 |


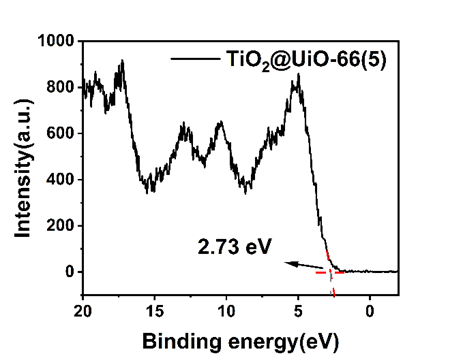


**Fig. S7** VB-XPS spectra of TiO_2_@UiO-66(5).
